# Circular Trajectory Reconstruction Uncovers Cell‐Cycle Progression and Regulatory Dynamics from Single‐Cell Hi‐C Maps

**DOI:** 10.1002/advs.201900986

**Published:** 2019-09-30

**Authors:** Yusen Ye, Lin Gao, Shihua Zhang

**Affiliations:** ^1^ School of Computer Science and Technology Xidian University Xi'an 710071 Shaanxi China; ^2^ NCMIS CEMS RCSDS Academy of Mathematics and Systems Science Chinese Academy of Sciences Beijing 100190 China; ^3^ School of Mathematical Sciences University of Chinese Academy of Sciences Beijing 100049 China; ^4^ Center for Excellence in Animal Evolution and Genetics Chinese Academy of Sciences Kunming 650223 China

**Keywords:** cell cycle, chromosomal architectures, circular trajectory, dynamic structures, single‐cell Hi‐C maps

## Abstract

Single‐cell Hi‐C technology is emerging and will provide unprecedented opportunities to elucidate chromosomal dynamics with high resolution. How to characterize pseudo time‐series of single cells using single‐cell Hi‐C maps is an essential and challenging topic. To this end, a powerful circular trajectory reconstruction tool CIRCLET is developed to resolve cell cycle phases of single cells by considering multiscale features of chromosomal architectures without specifying a starting cell. CIRCLET reveals its best superiority based on the combination of one feature set about global information and another two feature sets about local interactional information in terms of designed evaluation indexes and verification strategies from a collection of cell‐cycle Hi‐C maps of 1171 single cells. Further division of the reconstructed trajectory into 12 stages helps to accurately characterize the dynamics of chromosomal structures and explain the special regulatory events along cell‐cycle progression. Last but not the least, the reconstructed trajectory helps to uncover important regulatory genes related with dynamic substructures, providing a novel framework for discovering regulatory regions even cancer markers at single‐cell resolution.

## Graphical abstract

CIRCLET first extracts multiscale features of chromosomal architectures for each cell, and then reconstructs a circular trajectory of single cells. Further division of the reconstructed trajectory helps to characterize the dynamics of chromosomal structures and uncover important regulatory genes along cell‐cycle progression. CIRCLET is the first tool for accurate reconstruction of a pseudotime trajectory based on single‐cell 3D genome maps.

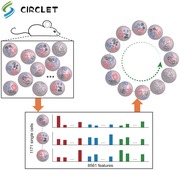

## 1 Introduction

Cellular processes such as cell proliferation, differentiation, and cycle are driven by complex gene regulatory programs, which often lead to strong cell‐to‐cell variability.^1^, ^2^, ^3^ Traditionally, a population of cells measured at the same time contains different types or states of cells, which would mask trends occurring among individual cells and fail to capture cellular dynamics and specificities. However, emerging single‐cell technologies referring to transcriptomics, genomics, metabolomics, chromatin accessibility, methylome, and 3D chromatin architecture have matured to facilitate a large number of cells in parallel at single‐cell resolution.^2^, ^4^, ^5^, ^6^, ^7^ Therefore, they provide unprecedented opportunities for researchers to leverage the continuum of transitional states of single cells and reveal their temporal and spatial variations directly.

Characterization of cellular heterogeneity and developmental trajectory is a challenging topic due to high variability between individual cells in biological processes, which could play important roles in treating cancer and other diseases,^8^ understanding the process of cellular aging,^9^ unraveling the mechanisms of gene regulation and expression programs, and so on. Computational methods for pseudo‐trajectory reconstruction have demonstrated that aligning cells is a quantitative and effective way to define dynamic progresses of cells based on single‐cell RNA‐seq data or mass cytometry.^10^, ^11^, ^12^, ^13^, ^14^, ^15^, ^16^ For example, Monocle uses independent component analysis to reduce the dimensionality of this space, followed by constructing minimum spanning trees (MST) and finding the longest path to produce a trajectory of cell differentiation of primary human myoblasts.^14^ TSCAN adopts a cluster‐based MST to reduce the complexity of tree space and order cells by mapping them to the edges between cluster centers.^13^ Wanderlust and Wishbone take a graph‐based algorithm to construct a robust distance metric and order cells to form a unified or branching trajectory.^3^, ^15^ Besides, compared with the branching process of cellular differentiation, some approaches have been developed to detect circular patterns of cells, such as cell cycle. ReCAT models the time‐series construction of cell cycle as a traveling salesman problem, and finds a shortest cyclic pattern through all cells.^12^ These methods have revealed that pseudo‐trajectory reconstruction is an effective way to study the dynamics of biological processes within the nucleus using single‐cell RNA‐seq data at transcriptomic level. Naturally, cellular trajectory reconstruction of single cells employing other single‐cell omics data is also expected.

Very recently, the progress of single‐cell Hi‐C using flow cytometry sorting allows us to develop computational methods to determine cycle phases of single cells and analyze the dynamics of chromosomal structure and organization.^2^ This pioneering study suggests to use four metrics of each individual cell to empirically divide single cells into five cycle phases through several specified thresholds and orders them in different phases by the values of one or two metrics defined above. However, this method oversimplifies the topological characteristics of the Hi‐C data, and is hard to be applied to single‐cell Hi‐C data of other cellular system directly. Moreover, it heavily subjects to diverse empirical thresholds, which are challenging for biological users to determine.^2^ Thus, efficient and automatic computational methods for exploring the dynamic characteristics of chromosomal architecture based on single‐cell Hi‐C data solely are urgently needed.

To this end, we develop a powerful and robust circular trajectory reconstruction tool CIRCLET without specifying a starting cell for resolving cell‐cycle phases of single cells considering multiscale features of chromosomal architectures. CIRCLET reveals its best superiority based on the combination of a feature set about global information and another two feature sets about local interactional information in terms of designed evaluation indexes and verification strategies from a collection of cell‐cycle Hi‐C maps of 1171 single cells. Further division of the reconstructed trajectory into 12 stages helps to accurately characterize the dynamics of insulation strength and compartments along cell‐cycle progression, suggesting that the compartments and topologically associated domains (TADs) are not a hierarchy of the same phenomenon at different scales, and may compete with each other during S phase. More prominent loops of TAD level can be observed during both G1 and early‐S (ES) phases than other phases accompanying with the strongest insulation strength, suggesting that architectural loops may drive the development of high‐level structures. Moreover, the reconstructed trajectory helps to reveal more phase‐specific regulatory loops associated with cell‐cycle checkpoint from G1 to ES phase, and rarely new loops associated with specific regulatory events of cell cycle in G2 phase. Finally, the reconstructed trajectory also helps to discover important regulatory genes related with dynamic substructures, providing a novel framework for discovery of regulatory regions even cancer markers at single‐cell resolution.

## 2 Results

### 2.1 Overview of CIRCLET

**Figure 1:**
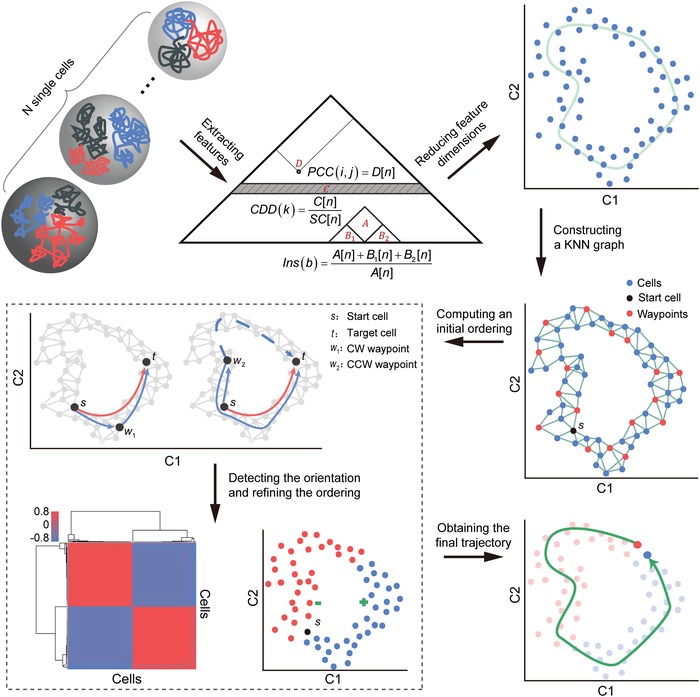
Illustration of CIRCLET for reconstructing a cell‐cycle trajectory from single‐cell Hi‐C maps. CIRCLET contains six key steps. 1) Extracting features: multiscale feature sets are extracted from single‐cell Hi‐C maps. 2) Reducing feature dimensions: the dimension of these feature sets are further reduced to a low *n*‐dimensional space via diffusion maps (e.g., 2D as an example). 3) Constructing a KNN graph: CIRCLET constructs a *k*‐nearest‐neighbor graph in the *n*‐dimensional embedded space and selects a set of cells called “waypoints,” one of which is randomly selected as the starting cell *s*. 4) Computing an initial ordering: an initial ordering of cells is obtained by the shortest path distance from *s* (e.g., distance *D*_*s*,*t*_ marked by a red solid line from *s* to *t* cell). 5) Detecting the orientation and refining the ordering: CIRCLET also computes a perspective matrix **P**, which records the shortest path distance of each cell to the starting cell *s* from the viewpoint of waypoints (e.g., the distance of cell *t* to *s* from the viewpoint of *w*_1_ is $P_{w,t}=D_{s,w_{1}}\;+\;\;D_{w_{1,t}}$). These waypoints' perspective is first used to identify the clockwise (CW) or counterclockwise (CCW) semicircle of cells from *s*. 6) Obtaining the final trajectory: CIRCLET iteratively executes step (5) until convergence, eventually obtaining a high‐resolution cell‐cycle trajectory.

CIRCLET assumes that 1) single‐cell Hi‐C maps can well describe the entire cell‐cycle progression, and 2) chromosomal structure is continuously changing along cell‐cycle progression from the global view. CIRCLET not only considers the contact probability profile, but also captures multiscale structural information of observed cells (**Figure** **1** and the Experimental Section). These feature sets can be summarized as a high‐dimensional vector for each single cell. As suggested by Wishbone for single‐cell RNA‐seq data,^3^, ^15^ CIRCLET applies a nonlinear dimensionality reduction method diffusion maps to generate a low‐dimensional embedding of high‐dimensional space and construct a *k*‐nearest‐neighbor (kNN) graph, where each node represents a cell and edges connect each cell to its *k* closest cells in the graph^17^ (Figure 1). This captures the major structure information of data to reduce existing measurement noise, thus dramatically reduce spurious edges. Note that Wishbone was designed for positioning single cells along bifurcating development trajectories, while CIRCLET aims to reconstruct circular time‐series of single cells by dividing it into two semicircle trajectories.

CIRCLET randomly assigns a starting cell, and generates an initial ordering of cells by considering the shortest path distance from the starting one. Since the trajectory is circular, the order of cells from the starting cell to cells in clockwise (CW) and counterclockwise (CCW) directions are intertwined in the initial ordering. CIRCLET uses a set of cells, called waypoints, to cleverly divide a circular trajectory into two semicircles, and refines the two subtrajectories, respectively (see the Experimental Section). Moreover, the shortest path distance deviates more and more with the distance to the target point increasing. CIRCLET refines the ordering of cells by a weighted average of distance to the target cell from all waypoints' perspective in the same semicircle trajectory (see the Experimental Section). CIRCLET iteratively performs the procedure of detecting orientation and refining ordering until convergence and finally generates a robust cell‐cycle trajectory.

### 2.2 The Reconstructed Trajectories of Four Individual Feature Sets and Their Combinations Show Diverse and Complementary Performance

Single‐cell Hi‐C maps of mouse embryonic stem cells (ESCs) are employed to make systematic exploration and illustration about how CIRCLET reconstructs a cell‐cycle trajectory with high resolution. Nagano et al.^2^ suggested to use four metrics of each individual cell: the percentage of near and mitotic contacts, mean contact distance of the far‐end interaction (>4.5M) and the fraction of early‐replicating fragment ends (fends) out of all fends, and empirically divide single cells into five cycle phases through several specified thresholds and order them in different phases by the values of one or two metrics defined above. However, this method oversimplifies the topological characteristics of the Hi‐C data and is hard to be applied to other Hi‐C data due to diverse empirical thresholds in different steps.

**Figure 2:**
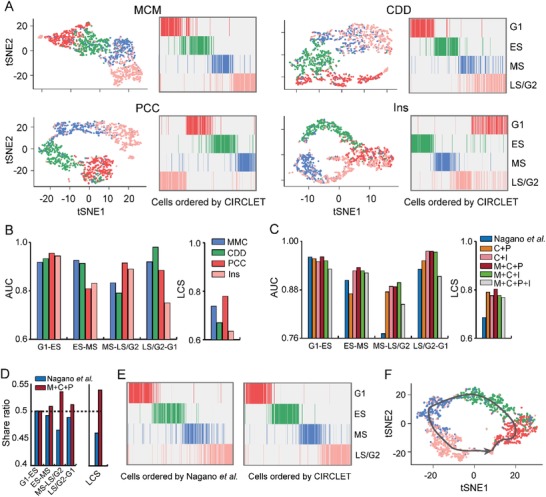
Exploring the preference of multiple feature sets and their combinations. A) tSNE maps and cell heatmaps of four FACS‐sorted cell phases (G1, ES, MS, and LS/G2) ordered based on the reconstructed trajectory by CIRCLET based on four individual feature sets including MCM (M), CDD (C), PCC (P), and Ins (I), respectively. B) Comparison of five evaluation indexes for the reconstructed trajectory by CIRCLET based on the four individual feature sets, respectively. These evaluation indexes include AUC scores between two successive cell‐cycle phases (denoted as G1–ES, ES–MS, MS–LS/G2, and LS/G2–G1) and LCS for measuring labels inconsistency of adjacent cells on the entire inferred trajectory. C) Comparison of the five evaluation indexes for the reconstructed trajectory by CIRCLET by Nagano et al. and five combinations of multiple feature sets. D) The share ratio of five scores between the inferred trajectories by Nagano et al. and CIRCLET based on the best combination of feature sets. E) The reconstructed cell‐cycle trajectories by Nagano et al. (left panel) and CIRCLET (right panel), respectively, based on the best combination (MCM+CDD+PCC) from four FACS‐sorted cells (G1, ES, MS, and LS/G2). F) tSNE maps by CIRCLET based on the best combination (MCM+CDD+PCC) from four FACS‐sorted cell phases (G1, ES, MS, and LS/G2).

Here we design four different feature sets: multiple composite metrics (MCM), contact probability distribution versus genomic distance (CDD), pairs' contact coverage (PCC), and insulation score of each bin (Ins), and their combinations as input of CIRCLET. Evaluation of their performance is conducted based on the known attributes of cells (G1, ES, mid‐S (MS), and late‐S/G2 (LS/G2)) labeled by fluorescence‐activated cell sorting (FACS) (see the Experimental Section). The reconstructed trajectory based on MCM reveals that the ordering between the boundaries of multiple phases is not captured well, but the overall ordering is globally valid (**Figure** **2**A,B), suggesting that MCM is globally informative. Moreover, it fails to form a cyclic trajectory. The reconstruction based on CDD fails to distinguish MS and LS/G2 phases well, indicating the change of contact probability distribution is relatively small, though the change in chromatin structure is relatively obvious during S phase due to DNA replication (Figure 2A,B). PCC representing loop signal possesses abundant cell‐cycle information to distinguish cells at different phases, but there are many outliers which are significantly deviated from original phases (Figure 2A,B). This may be due to the fact that PCC collects valid details but lacks macro information. Ins does not make a good distinction between LS/G2 and G1 (Figure 2A,B), which is associated with the loss of compartments and TADs in prophase, and these structures start appearing again in G1 phase.^18^

In brief, CDD captures contact probability profile, PCC and Ins record higher‐order structural properties, and MCM suggests macro information of genomic architecture. The reconstructed trajectory based on the combination of CDD and Ins is superior to the one based on the combination of CDD and PCC (Figure 2C). The reconstructed trajectories show that the combination of MCM, PCC, and CDD obtains the best performance (Figure 2C), suggesting that the three sets of features can be properly complementary to each other. However, the combination of four feature sets does not perform better, and it may be correlated with signals from PCC and Ins that weaken each other (Figure 2C). In summary, all five scores of the inferred trajectory by CIRCLET based on the combination of MCM, PCC, and CDD feature sets outperforms those of Nagano et al.^2^ (Figure 2C–F). The sum of increased ratio of five scores is ≈36% (Figure S1A, Supporting Information), demonstrating its superiority to the original study. Thus, we suggest to use the combination of three feature sets (MCM, PCC, and CDD) to reconstruct the trajectory.

We also examined that CIRCLET is capable of reconstructing cyclic trajectories robustly by deleting some cells, or filling some cells along circular trajectory (Figure S1B and Methods, Supporting Information). CIRCLET shows distinct robustness compared to the simple metrics used by Nagano et al.^2^ in both two cases (Figure S1C, Supporting Information).

### 2.3 The Reconstructed Trajectory Helps to Discover the Dynamic Chromatin Substructures

**Figure 3:**
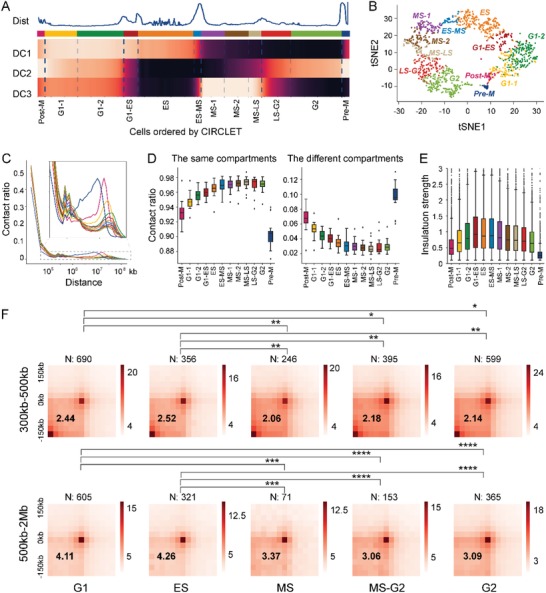
The reconstructed trajectory helps to reveal accurate dynamics of chromatin substructures. A) The reconstructed trajectory is divided into 12 stages based on the top three diffusion components. B) tSNE map of the single cells marked by different color indicating different stage labels. C) Contact probability in logarithmic bins based on pooled Hi‐C maps at 40 kb resolution across 12 stages. D) Contact probability between the same compartments (“A” vs “A” and “B” vs “B”) and different ones (“A” vs “B”) based on pooled Hi‐C maps at 100 kb resolution across 12 stages. E) Insulation strength of TAD boundaries at 40 kb resolution across 12 stages. F) Average Hi‐C maps around loops of different distance (300–500 kb and 500 kb to 2M) in five merged stages within ±150 kb based on the pooled Hi‐C maps at 25 kb resolution. These subcycles include G1 (G1‐1 and G1‐2), ES (ES), MS (MS‐1 and MS‐2), MS–G2 (MS–LS and LS–G2), and G2 (G2). The enrichment of a set of loops displayed in the lower left corner of heatmap evaluated by the ratio of the central pixel to the mean of the pixels in the lower left corner on the above average matrix. The comparison between two loop sets is calculated by Wilcoxon rank‐sum statistic (*P < 10^−2^, **P < 10^−5^, ***P < 10^−8^, ****P < 10^−11^).

We divide the reconstructed trajectory into 12 different stages to better capture the dynamic characteristics of chromatin structures, and the same stage of cells have very similar components of diffusion maps (**Figure** **3**A and the Experimental Section). tSNE map shows the division accurately aggregate similar single cells (Figure 3B). Thus, we pool the Hi‐C maps of all cells in the same stage together to generate an aggregated Hi‐C map for each stage.

The analysis of contact probability along interaction distance shows a global reorganization of chromatin structures during cell cycle (Figure 3C; Figure S2, Supporting Information). The short‐range contacts (200 kb to 2 Mb) gradually increases, while long‐range contacts (greater than 5 Mb) is opposite until Pre‐M phase (Figure 3C). Pre‐M phase reveals a characteristic scale of contact distances peaking between 2 Mb and 12 Mb, which is consistent with the observation for M phase cells in the bulk Hi‐C analysis.^19^

The compartment A/B identified based on the eigenvector value and the TADs identified based on the insulation score among the 12 stages both show distinct dynamic changes (Figure 3D,E). Obviously, the contact fraction between the same compartments increases, and the fraction between different compartments is opposite, until MS–LS phase. The insulation strength across TAD boundaries reaches the maximum in G1–ES phase,^20^ and after G1–ES phase, contacts across TAD boundaries begin to increase (see the Experimental Section). These results are consistent with previous studies, but more accurately specify substages of functional or structural transitions, and more specifically characterize the dynamics of cell cycle.^2^ Generally, TADs show the clearest segmentation in G1–ES phase that is at the beginning of DNA replication, while compartmentalization increases until MS–LS phase that is at the end of DNA replication. Therefore, the compartments and TADs are not a hierarchy of the same phenomenon at different scales and may compete with each other during S phase.^21^

We further merge similar stages above to obtain five larger ones with higher resolution Hi‐C maps for chromatin loop detection (see the Experimental Section). Obviously, both G1 and G2 phases are two substages obtaining a greater number of loops, which may be due to requirement for activated transcriptions and regulations for cell growth in these two phases (Figure 3F; Table S1, Supporting Information). G1 phase performs cell growth in size and ensures everything for DNA synthesis and G2 phase is a period of rapid cell growth and protein synthesis during which the cell prepares itself for mitosis. However, S phase is the period of DNA replication, and rates of RNA transcription and protein synthesis are low during this phase.

It can be observed that the chromatin loops of both G1 and ES phases are more prominent compared with three other phases (Figure 3F and the Experimental Section). Furthermore, the difference is more apparent on loops of long‐range (500 kb to 2 Mb) than those of short‐range (300–500 kb) (Figure 3F). We guess that many architectural loops related with TADs are formed between G1 and ES phases. This phenomenon agrees with the strongest insulation across TAD boundaries during this phase as above. These results suggest that the formation of chromatin loops may drive the development of high‐level structures (e.g., TADs).^21^, ^22^, ^23^

### 2.4 The Reconstructed Trajectory Helps to Explain Regulatory Events of Dynamic Chromatin Substructures

**Figure 4:**
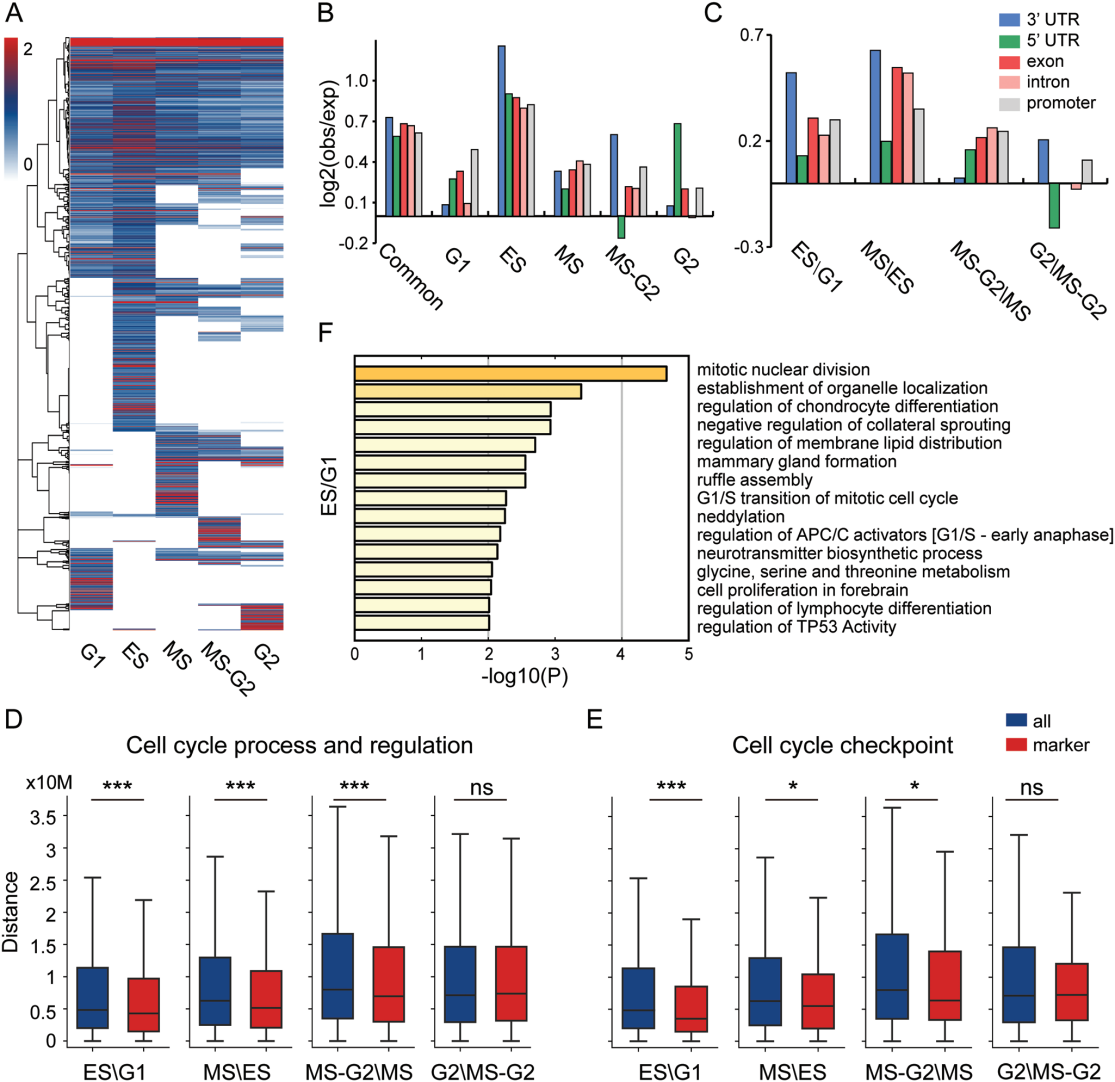
Interpretation of regulatory events of dynamic chromatin substructures. A) Hierarchical clustering of TAD boundaries with high confidence based on insulation strength across five substages. B) Enrichment of overlapping between TAD boundaries and genomic features. The boundaries consist of common and stage‐specific ones. C) Characterization of differential loops relative to different genomic features. Differential loops are detected between two continuous stages. These loops appear in the first loop list, but not in the second loop list (e.g., appearing in ES not in G1 for ES\G1). D,E) The distance distribution from background genes (all) and two annotated gene sets (marker) to differential loop lists between two continuous subcycles. Statistical significance is calculated by Wilcoxon rank‐sum statistic (*P < 0.05, **P < 0.01, ***P < 0.001, ns, not significant). F) Functional enrichment analysis of genes overlapping with differential loops between ES and G1.

TAD boundaries of high confidence with a uniform threshold across cell‐cycle progression were kept for detailed analysis. Obviously, ES phase contains significantly more high‐confident boundaries and overlapping genes than other phases (Table S2, Supporting Information; see the Experimental Section). More than 22% of these boundaries are common across the whole cell cycle, and nearly 34% of them are cycle‐specific (**Figure** **4**A). Interestingly, common boundaries are enriched in gene regions, and the enrichment of specific ones in gene regions changes dynamically along cell‐cycle progression. The enrichment of ES phase is significantly larger than that of other phases, even common boundaries (Figure 4B), which may be associated with the strongest intra‐TAD interactions and special regulations before DNA replication.^24^

New formation and disappearance of loops along cell‐cycle progression can be observed (Tables S3 and S4, Figure S3A,B, Supporting Information; see the Experimental Section). Obviously, MS phase forms more new loops related with gene regions than other phases (Figure 4C). We can see that that new loops formed in G2 phase are weakly related with 3′ untranslated region (UTR) and promoters, which may result from preparation of the regulation for G2 phase completed in transition phase (MS–G2 phase) or experimental noise caused by DNA concentration in G2 phase.

New loops appearing in ES\G1, MS\ES, and MS–G2\MS are strongly associated with cell‐cycle annotated genes in both two lists than G2\MS‐G2 (Figure 4D). Moreover, new loops appearing in ES\G1 are more strongly related with cell‐cycle checkpoint annotated genes than others (Figure 4E). These results suggest that many specific regulatory loops associated with cell‐cycle checkpoint are generated from G1 to ES phase and G2 phase rarely formed new loops associated with specific regulatory events of cell cycle.

The newly formed loops during G1 to ES phase are mainly associated with two major categories (Figure 4F; details in Figure S4A and Table S5, Supporting Information). One controls regulatory events of G1/S phase transition, including regulation of checking points, APC/C activators, p53 activity, etc. More specifically, checkpoints prevent cell‐cycle progression at specific transition points, allowing verification of necessary phase processes and repair of DNA damage.^25^ APC/C activator protein is thought to prevent premature S‐phase entry by degrading mitotic cyclins in G1 phase.^26^ p53 plays an important role in triggering the control mechanisms at G1/S checkpoints.^27^ Another one is related with the establishment of organelle localization, the regulation of membrane lipid distribution, and ruffle assembly, which may go along cell size growth in G1 phase and have to reorganize intracellular membrane distribution. These loop‐associated genes form a densely connected network components, which is enriched in the regulation of cell cycle and cell‐cycle checkpoint (Figure S4B, Supporting Information). More analysis of cell‐cycle transitions can be seen in Tables S6–S8 in the Supporting Information.

### 2.5 The Reconstructed Trajectory Helps to Discover Important Regulatory Genes at Single‐Cell Resolution

**Figure 5:**
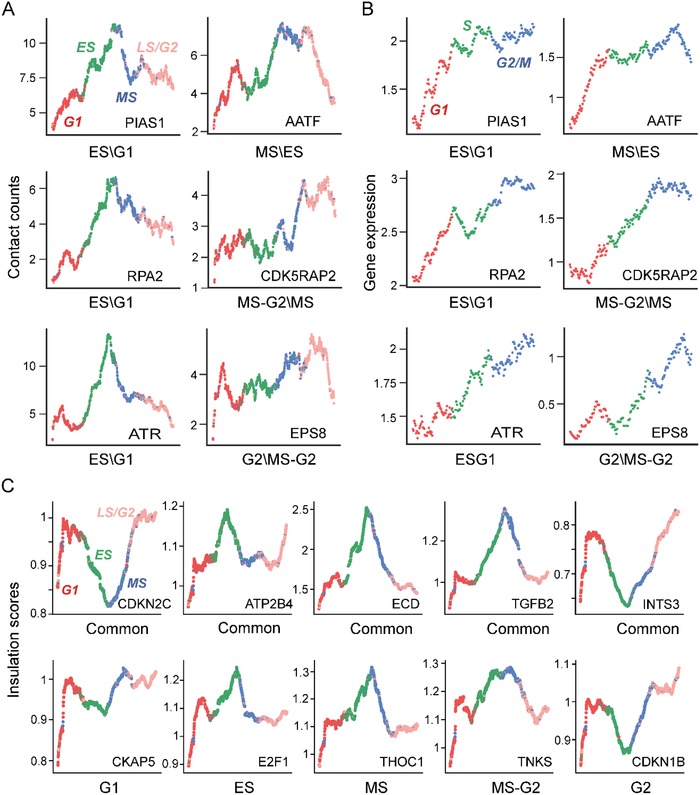
Case studies of important regulatory genes at single‐cell resolution. A) Smoothed contact counts of differential loops overlapping with important regulatory genes across cell‐cycle progression at single‐cell resolution. The first subgraph demonstrates the four cycle phases of single cells with different colors (red: G1, green: MS, blue: MS, and pink: LS/G2). B) Smoothed gene expression corresponding to (A) across cell‐cycle progression at single‐cell resolution. The first subgraph demonstrates the three cycle phases of single cells with different colors (red: G1, green: S, and blue: G2/M). C) Smoothed insulation score of common and specific TAD boundaries overlapping with important regulatory genes across cell‐cycle progression at single‐cell resolution. The colors of cycle phases are consistent with (A) in the first subgraph.

The reconstructed trajectories from single‐cell Hi‐C maps and a single‐cell RNA‐seq dataset enable us to investigate the regulatory mechanism of differential loops along phase transition process (Figure S5, Supporting Information). Specially, we can clearly see that a new loop whose anchors overlaps with PIAS1 gene gradually formed from G1 to the end of ES phase (**Figure** **5**A). It has been suggested that this gene is a checkpoint regulator affecting exit from G1 through binding and sumoylation of p73,^28^ and its gene expression is increasing from G1 to S phase (Figure 5B). Other two loops formed in the end and middle of ES phase, and the anchors of these two loops overlap with RPA2 and ATR genes, respectively (Figure 5A). The contact count of one of these two loops rapidly decreases coupled with continuous increase of the expression level of ATR, and the other decelerates slowly with cell‐cycle progression coupled with expression increase of RPA2 after ES phase (Figure 5A,B). As we know that ATR is an essential kinase activated during S phase to regulate the firing of replication origins^29^ and RPA2 phosphorylation plays a critical role in maintenance of cell survival after a DNA replication during S phase,^30^ indicating the loop forming could play key roles in their regulation. In addition, RPA2 is a direct downstream target for ATR to regulate the S‐phase checkpoint.^31^ Thus, the dynamics of these two loops inferred based on the trajectory helps to reveal that RPA2 and ATR may cooperate to complete important regulatory events from G1 to ES phase transition and DNA replication during S phase.

AATF overlaps with a loop anchor, and this loop appears at the middle of MS phase and then continues to maintain until the beginning of G2 phase (Figure 5A). It plays an important role in the DNA damage response and cell‐cycle checkpoint control, and is originally characterized as an interacting protein for RNA polymerase II connecting transcriptional regulation. Thus, AATF may promote phase transition during S phase and perform transcriptional regulation for cell growth during G2 phase,^32^ which are also consistent with the increased expression of AATF gene during both S and G2 phases (Figure 5B).

CDK5RAP2 encoding a regulator of CDK5 activity plays an important role in spindle checkpoint activation, and promotes microtubule polymerization, bundle formation, growth, and regulation of centrosomal maturation.^33^ It overlaps with the anchor of a loop formed from MS to MS–G2 phase (Figure 5A). Moreover, the expression level of CDK5RAP2 gene peaks at the beginning of G2 phase (Figure 5B). These observations are consistent with the basic activities of the G2 phase, including protein synthesis, rapid cell growth, and microtubules begin to reorganize to form a spindle. EPS8 as part of the EPS8–IRSp53 complex mediating the regulation of cell shape by CDC42^34^ overlaps with the anchor of one loop formed from MS–G2 to G2 phase (Figure 5A), and it is also highly expressed during G2 phase (Figure 5B). It links to a loop forming, indicating its strong relevance with the preparation for separation of sister chromatids.

We find that insulation strength of common TAD boundaries undergoes significant dynamic changes with cell‐cycle progression, and different boundaries perform differential dynamic‐modes (Figure 5C). For example, the boundary overlapping with CDKN2C gene is the most prominent one in G1 and G2 phases (Figure 5C). CDKN2C has been shown to interact with CDK4 or CDK6, and prevent the activation of the CDK kinases, thus function as a cell growth regulator that controls cell‐cycle G1 progression. CDKN2C may also exercise specific regulation in G2 phase. In addition, the boundary overlapping with ECD gene is the most prominent one in ES phase (Figure 5C), and the evidence suggests that ECD‐deleted cells show a delay in G1‐S cell‐cycle progression with a delay in Rb (retinoblastoma) phosphorylation and reduce expression of E2F target genes.^35^

We find that ES‐phase‐specific boundaries interact with E2F1 gene (Figure 5C), which is a member of the E2F family of transcription factors and plays a crucial role in the control of cell cycle from G1 to S phase.^36^ Furthermore, CDKN1B with high insulation strength in G1 and G2 phases, is a cyclin‐dependent kinase inhibitor that blocks the cell cycle in the G1 phase upon differentiation signals or cellular insult (Figure 5C). Recently, researchers uncover a novel function of CDKN1B in the adult hippocampus as a dual regulator of stem cell quiescence and of cell‐cycle exit of immature neurons, which match well with our observation on insulation strength in G2 phase.^37^ Other analysis of cell‐cycle regulatory genes overlapping with stage‐specific loops, common, and specific TAD boundaries can be seen in Tables S12 and S13 in the Supporting Information. In short, the above results suggest that specific loops, common, and specific TAD boundaries are indeed associated with cell‐cycle‐specific regulatory events, providing a promising way to explore functional regions and regulatory markers at a special and exhaustive period at single‐cell resolution.

## 3 Conclusion

We have developed a powerful and robust tool CIRCLET for accurate reconstruction of circular trajectory with high resolution. We illustrate its effectiveness and robustness on resolving cell‐cycle progression using a collection of large‐scale single‐cell Hi‐C maps. We extract four different types of feature sets from these Hi‐C maps and analyze their characteristics on cell‐cycle ordering by examining their exclusivity and complementarity. CIRCLET demonstrates distinct superiority to a naïve strategy in terms of designed evaluation indexes and verification strategies from different perspectives.

We divide the reconstructed trajectory into 12 different stages based on the top three diffusion components. Further analysis suggests that cell cycle is accompanied by a global chromatin reorganization. Obviously, cell cycle goes together with an increasing compartmentalization until MS–LS phase, and an increasing insulation strength before G1–ES phase but a gradual decrease after this phase. The reconstructed trajectory could more accurately and specifically characterize the dynamics of cell cycle. Moreover, dynamic changes of loops are related to the formation of TADs, suggesting its drive force to the development of high‐level structures.

Examination of stage‐specific loops, common, and specific TAD boundaries along cell‐cycle progression demonstrates more detailed findings. For example, MS phase forms more new loops related with gene regions than other phases and many regulatory loops associated with cell‐cycle checkpoint are generated from G1 to ES phases. Further functional enrichment analysis on genes overlapping with anchors of new loops during G1\ES phase transition reveals two types of associated functions—regulatory event of G1/S phase transition and reorganization of intracellular membrane distribution, which are just the major biological processes carried out in G1/S phase.

Finally, the reconstructed trajectory can help to discover important regulatory genes at single‐cell resolution especially the stage‐specific regulatory ones. Some of these specific cell‐cycle regulatory genes are associated with the inhibition and induction of cancer, which suggests that dysregulation of genes in these regions may induce or inhibit tumor during cell‐cycle progression. For example, THOC1 inhibits cell growth via induction of cell‐cycle arrest and apoptosis in lung cancer cells.^30^ PTEN was identified as a tumor suppressor and was mutated in a large number of cancers at high frequency, including breast, prostate, endometrium, ovary, colon, melanoma, glioblastoma, and lymphoma cancers.^31^, ^32^ This evidence implicates the use of TNKS inhibitors to target the Wnt pathway to combat lung cancer.^38^ These observations may prove to be clinically valuable for developing a new therapeutic target of cancer. Thus, this analysis provides a framework for the subsequent discovery of important regulatory regions even cancer targets of special cycle activation at single‐cell resolution. With deeper single‐cell Hi‐C technology applied to human tissues, CIRCLET will further allow us for a deeper understanding of relationship between genome architecture and regulatory programs.

Our study provides a computational and analytical framework to advance our understanding of chromatin organization at single‐cell resolution across cell‐cycle progression. Compared with the method of Nagano et al., CIRCLET can accurately and naturally divide the trajectory into more substages, which could help us analyze substructures of chromatin and discover important regulatory genes in more detail. With rapid accumulation of single‐cell datasets, CIRCLET is expected to play vital roles in revealing detailed cellular functions and biological processes. However, there are still four urgent challenges needed to be addressed in future studies. First, a large number of dropouts in single‐cell Hi‐C maps make it ambiguous to explain dynamic changes of chromosome substructures. Second, it is necessary to develop a multiview model to explain cell‐cycle chromosomal dynamics with other biological processes, such as differentiation. Third, it remains an open question on how to better integrate multiomics data of single cells. Forth, the phase transition of some regions can result in discontinuous change locally,^39^ but should have no distinct effect to the construction of continuous trajectories from the global view.

## 4 Experimental Section

*Datasets*: The single‐cell Hi‐C dataset used in this study consists of 1992 diploid cells of mouse embryonic stem cells grown in 2*i* media without feeder cells with stringent quality control filter.^40^ This dataset involves in a median number of 393506 restriction fragments, and 127233 distinct >1 kb contacting pairs on average per cell. The cell‐cycle stages of single cells were examined by fluorescence‐activated cell sorting sort criterion. Based on it, 280, 303, 262, 326, and 401 cells belong to “G1” phase, “early‐S” phase, “mid‐S” phase, “late‐S/G2” phase, and “2*n* DNA” stage, respectively.

A single‐cell RNA‐seq data of mouse ESCs labeled by FACS sort criterion of 182 cells was also downloaded, including 59, 58, and 65 cells belonging to “G1” phase, “S” phase, and G2M phase, respectively.^41^ A set of 959 annotated genes of cell cycle was collected for analysis with variation above the background level.^41^

*CIRCLET*: CIRCLET extracts a set of features from the Hi‐C map for each cell at first. Then, CIRCLET constructs a *k*‐nearest‐neighbor graph of cells in the *n*‐dimensional space embedded by diffusion maps and estimates distances between them using the shortest path distance as described by Wishbone.^3^ CIRCLET selects a series of cells called waypoints along the whole trajectory to provide sparse approximation for the entire dataset, and randomly specify one of them as the starting cell. An initial ordering of cells was determined by the shortest path distances from the starting cell *s*. CIRCLET also computes a so‐called perspective matrix recording the shortest path distance of each cell to the starting cell *s* from the viewpoint of these waypoints. Furthermore, disagreements between the perspectives of waypoints were used to split the cyclic trajectory into two semicircles of opposite directions. The challenge of circular trajectory ordering was to transform it into two nonbranch trajectory inference problems in CW or CCW directions. Since the initial ordering of cells was more susceptible to noise as the distance increases, waypoints were also used to locally refine the ordering of cells in the same direction. The procedures of detecting orientation and refining ordering were iteratively repeated until convergence. CIRCLET is described in detail as follows.

*CIRCLET—Extracting Features*: There exist many random interacting contacts, technical noise, and dropout events among the single‐cell Hi‐C maps. It is hard to directly use the very high‐dimensional and sparse data. Feature extraction is essential to extract interesting biological signals and reduce the computational complexity for downstream analysis. To this end, features were extracted in the following four ways.

*CIRCLET—Extracting Features—Contact Probability Distribution versus Genomic Distance*: The distribution of the Hi‐C contacts was calculated in log2 genomic distance bins. Specifically, the following formula was defined to calculate log2 distance of each fragment pair

(1) $$\mathrm{loc}=\mathrm{floor}\left(\frac{\log_{2}\left(d\right)+s}{s}\right)$$

where *d* indicates the genomic distance of fragment pairs (*d* > 20 kb) and *s* means an exponent step of each bin, whose typical values are [0.1, 0.125, 0.2, and 0.33]. The count of fragment pairs per bin divided by the count of fragment pairs for all bins was defined as contact probability of the bin. According to different span of steps, four different scales of feature sets were obtained for the contact probability profile.

*CIRCLET—Extracting Features—Pairs' Contact Coverage*: Intrachromosomal contacts of a single‐cell Hi‐C map were typically summarized as a matrix *A* with bins of a fixed width (e.g., 100 kb, 500 kb, 1 Mb) for each chromosome, in which *A_i,j_* (1 ≤ *i* ≤ *n*, 1 ≤ *j* ≤ *n*) represents the interacting frequency (or a normalized score) between bins *i* and *j*. $\mathrm{Value}(A_{i,j})=\frac{\sum_{k=1}^{k}A_{i,j,k}}{k}$ was used to characterize the interacting significance of *A_i,j_* across all single cells, where *k* means the *k*th cell and *K* represents the number of cells. $\mathrm{var}(A_{i,j})$ indicates the variance of *A_i,j_* across all single cells. Bin pairs with the top 99% of value(*A*_*i*,*j*_) were defined as significant ones.

*CIRCLET—Extracting Features—Insulation Score of Each Bin*: As described above, intrachromosomal contacts of a single‐cell Hi‐C map was typically summarized as a matrix *A* with bins of a fixed width for each chromosome. The insulation score of the *b*th bin was defined as $\mathrm{Ins}(b)=\sum_{i=b-\mathrm{scale}}^{b+\mathrm{scale}}\;\sum_{j=b-\mathrm{scale}}^{b+\mathrm{scale}}A_{i,j}/\sum_{i=b-\mathrm{scale}}^{b}\sum_{j=b}^{b+\mathrm{scale}}A_{i,j}$, where scale represents the size of the window. Furthermore, bins were selected with the average or variance of insulation score above the 90% quantile across all bins.

*CIRCLET—Extracting Features—Multiple Composite Metrics*: Multiple composite metrics proposed for trajectory inference or downstream analysis in the work by Nagano et al.^2^ were also considered as one type of feature set (Table S11, Supporting Information).

*CIRCLET—Reducing Feature Dimensions and Constructing a kNN Graph*: Inspired by Wishbone,^3^, ^13^ *N*‐dimension feature sets were first reduced into *M*‐dimensional space via diffusion maps. CIRCLET further constructs a kNN graph, **G**, in the embedded space where each node connected its *k* nearest nodes by Euclidean distance and edge weights are expressed by Euclidean distance between nodes.

*CIRCLET—Computing an Initial Ordering*: CIRCLET selects a series of cells called “waypoints” along the whole kNN graph and randomly specifies one of them as the starting cell *s*. An initial ordering of cells was determined by the shortest path distance from *s* to other cells. The shortest path distance between two cells was determined by Dijkstra's algorithm. The initial position of cell *i* along the trajectory was specified by the shortest path distance from *s*, expressed as $\tau_{i}^{\left(0\right)}=D_{s,i}$.

*CIRCLET—Detecting the Orientation and Refining the Ordering*: As the initial ordering of cells was more susceptible to noise as the distance increases from *s*, CIRCLET samples a series of waypoints to guide the cell ordering. CIRCLET selects waypoints by a median filter strategy to prevent the outlier cells from being chosen.^3^ Next, CIRCLET computes a perspective matrix $P\in {\mathbb{R}}^{W\;\times \;N}$, where *W* is the number of waypoints and *P_w,i_* represents the distance of cell *i* to the starting cell *s* from the viewpoint of waypoints *w*, defined as

(2) $$P_{w,i}=\{\begin{array}{c} \tau_{w}^{\left(0\right)}+D_{w,i}\;\;\;\;\;\;\;\mathrm{if}\;\tau_{i}^{\left(0\right)}>\tau_{w}^{\left(0\right)}\\ \tau_{w}^{\left(0\right)}-D_{w,i}\;\;\;\;\;\;\mathrm{otherwise} \end{array}$$

where *D_w,i_* is computed by the shortest path distance from waypoint *w* to cell *i*, the distance among all cells are represented by the matrix $D\in {\mathbb{R}}^{W\;\times \;N}$.

*CIRCLET—Detecting the Orientation and Refining the Ordering—Detecting the Orientation*: Different with Wishbone, CIRCLET first applies the perspective matrix **P** dividing the circular trajectory into two semicircles of opposite direction (CW or CCW) around the starting cell *s*. Considering two waypoints (*w*_1_ and target cell *t*) in the same semicircle, the shortest path distance from *s* to *t* roughly agrees with the distance used for calculating perspective of *t* from viewpoint of *w*_1_. That is, the difference between *D_s,t_* and $P_{w_{1},t}=D_{s,w_{1}}+D_{w_{1},t}$ is relatively a small number (≈0) (Figure 1). Now considering two waypoints (*w*_2_ and target cell *t*) from different semicircles, regardless of either the dashed or solid line in Figure 1, is the real shortest path from *s* to *t*, the difference between *D_s,t_* and $P_{w_{2},t}=D_{s,w_{2}}+D_{w_{2},t}$ is relatively large (>>0). Thus, the disagreement between the two semicircle waypoints' perspective relative to each other provides a quantitative measure of which semicircle waypoints lie on.

CIRCLET creates the disagreement matrix $Q\in {\mathbb{R}}^{W\;\times \;W}$ among all waypoints, where

(3) $$Q_{w_{i},w_{j}}=\left|P_{w_{i},w_{j}}-\tau_{w_{j}}^{\left(0\right)}\right|$$

Specifically, $Q_{w_{i},w_{j}}\approx 0$ indicates the two waypoints *w_i_* and *w_j_* are on the same semicircle trajectory, while $Q_{w_{i},w_{j}}\gg 0$ indicates they are on different semicircles. A natural idea was to adopt an unsupervised clustering method to split these waypoints into two categories of two semicircles. CIRCLET applies a hierarchical clustering method to the Pearson correlation coefficient matrix between rows or columns of **Q** matrix, and classifies these waypoints into sets in two different semicircles, **S**_1_ and **S**_2_. The remaining cells were assigned to the semicircle set of their nearest waypoints. The original challenge was transformed into two nonbranch trajectory inference problems in CW or CCW directions. The distance matrix **D** was divided into two blocks $D^{\left(S_{1}\right)}\in {\mathbb{R}}^{W^{\left(S_{1}\right)}\;\times \;N^{\left(S_{1}\right)}}$ and $D^{\left(S_{2}\right)}\in {\mathbb{R}}^{W^{\left(S_{1}\right)}\;\times \;N^{\left(S_{2}\right)}}$, where $W^{\left(S_{1}\right)}$ is the number of waypoints and $N^{\left(S_{1}\right)}$ is the number of cells in **S**_1_ semicircle, $W^{\left(S_{2}\right)}$ and $N^{\left(S_{2}\right)}$ are similar. Matrix **P** is similarly divided in two blocks $P^{\left(S_{1}\right)}\in {\mathbb{R}}^{W^{\left(S_{1}\right)}\;\times \;N^{\left(S_{1}\right)}}$ and $P^{\left(S_{2}\right)}\in {\mathbb{R}}^{W^{\left(S_{2}\right)}\;\times \;N^{\left(S_{2}\right)}}$.

*CIRCLET—Detecting the Orientation and Refining the Ordering—Refining the Ordering*: These waypoints were employed to robustly order the cells by computing a weighted average of distance to the starting cell *s* from their viewpoint in the same semicircle (e.g., CIRCLET refines *D_s,t_* using *w*_1_ not *w*_2_) (Figure 1). Next, the semicircle set **S**_1_ was taken as example to refine the ordering of cells in a nonbranch trajectory. The closer waypoints gave a bigger “vote” for the ordering of cells to take advantage of reliability of the shortest paths at shorter distance. Following the idea of Wishbone, CIRCLET calculates the weight of waypoint *w* by a Gaussian kernel applied to the distances of cell *i*, defined as

(4) $$W_{w,i}^{\left(S_{1}\right)}=\exp\left(\frac{-{\left(D_{w,i}^{\left(S_{1}\right)}\right)}^{2}}{\sigma^{\left(S_{1}\right)}}\right)/\sum_{k=1:N^{\left(S_{1}\right)}}\exp\left(\frac{-{\left(D_{w,k}^{\left(S_{1}\right)}\right)}^{2}}{\sigma^{\left(S_{1}\right)}}\right)$$

where $\sigma^{\left(S_{1}\right)}$ is the standard deviation of distance matrix $D^{\left(S_{1}\right)}$. The denominator is the summation of numerator over all cells and used for normalization. Thus, the refined trajectory of cell *i* in **S**_1_ is calculated by

(5) $$\tau_{i,S_{1}}^{\left(1\right)}=\sum_{w=1:W^{\left(S_{1}\right)}}P_{w,i}^{\left(S_{1}\right)}\times W_{w,i}^{\left(S_{1}\right)}$$

CIRCLET also computes the refined trajectory of all cells in **S**_2_ similarly. Merging the above two trajectories, CIRCLET obtains an updating trajectory vector τ^(1)^ for all cells.

*CIRCLET—Obtaining the Final Trajectory*: CIRCLET iteratively executes the above processes (step 5) until convergence: corr(τ^(*t*)^,τ^(*t* − 1)^) > 0.9999 . Finally, CIRCLET obtains two convergent trajectories $\tau_{S_{1}}^{\left(t\right)}$ and $\tau_{S_{2}}^{\left(t\right)}$ with orientation assigning sets **S**_1_ and **S**_2_, respectively. If **S**_1_ is set in CCW direction and **S**_2_ is opposite, then the output trajectory of CIRCLET is normalized to [0,1] from $\tau =\left[-\tau_{S_{1}}^{\left(t\right)},\tau_{S_{2}}^{\left(t\right)}\right]$. If the orientation of **S**_1_ and **S**_2_ is contrary to the above assumption, CIRCLET obtains a normalized output from $\tau =\left[\tau_{S_{1}}^{\left(t\right)},-\tau_{S_{2}}^{\left(t\right)}\right]$. Obviously, the two results are exactly the same circular trajectories that were expected.

In summary, CIRCLET aims to achieve high‐resolution ordering of single cells along cell‐cycle phases, which is generally expressed as a circular trajectory inference problem. First, CIRCLET extracts topological features from the single‐cell Hi‐C maps for each cell, and then constructs a kNN graph based on them and obtain the initial ordering of cells using the shortest path distance. Meanwhile, CIRCLET computes distance matrix **D** from waypoints to all cells. The processes were only performed once. Next, CIRCLET iteratively determines a perspective matrix **P** from matrix **D**, which in turn is used to calculate the disagreement matrix **Q** utilized to distinguish orientation and refine the ordering of cells, until it converges.

*Evaluation of the Reconstructed Cell‐Cycle Trajectory*: Two sets of quantitative measures were adopted to accurately quantify the consistency between the identified time‐series and the known cell‐cycle phase labels. CIRCLET outputs a time‐series between 0 and 1 (or ranking between 1 and *n*) for all single cells, which are partially labeled different phases by FACS experiment, including “G1,” “early‐S,” “mid‐S,” and “late‐S/G2” phases. Since cell cycle is circular, it is assumed that the inferred time‐series or the labeled phases are aligned end to end.

*Evaluation of the Reconstructed Cell‐Cycle Trajectory—Label Change Score (LCS)*: LCS measures frequency of change for single cells' labels determined by FACS experiment along the entire trajectory. LCS is defined by $\mathrm{LCS}=1-\frac{S_{c}-4}{N-4}$, where *s*_c_ is the number of changed labels between experimentally labeled cells along the trajectory and *N* is total number of experimentally labeled cells. Ideally, a perfect LCS score equals 1, and the worst score equals 0.

*Evaluation of the Reconstructed Cell‐Cycle Trajectory—Area under the Curve (AUC)*: AUC measures the agreement between the inferred time‐series and the known phases of single cells for any two successive phases. Specifically, one cell‐cycle phase was assumed as the positive class and its next phase as negative. According to the trajectory of single cells, the proportion of positive samples under different thresholds was calculated to form ROC curve and its corresponding AUC was obtained.

*Division of the Reconstructed Trajectory into Substages*: The top three diffusion components were first smoothed using the average of 11 single cells centered at each cell along the inferred trajectory (Figure 3A). Furthermore, Euclidean distance between adjacent cells was calculated to obtain a distance vector along the inferred trajectory, and the vector was further smoothed using the average of 11 scores centered at each value (Figure 3A). The location of phase transition was specified by checking the peak of the distance vector and further subdividing three of these phases according to split locations of progression change of the top three diffusion components (Figure 3A). Finally, the inferred trajectory was divided into 12 different substages, including Post‐M, G1‐1, G1‐2, G1–ES, ES, ES–MS, MS‐1, MS‐2, MS–LS, LS–G2, G2, and Pre‐M. The dynamics of compartments and TADs were further analyzed at 40 or 100 kb resolution among these substages. To detect chromatin loops and more specific TAD boundaries from Hi‐C maps at 25 kb resolution, similar substages were merged to generate five available larger substages, including G1 (G1‐1 and G1‐2), ES (ES), MS (MS‐1 and MS‐2), MS–G2 (MS–LS and LS–G2), and G2 (G2).

*Identification of Chromatin Substructures—Compartments*: Compartment A/B was identified by calculating the dominant eigenvector of intrachromosomal contacts matrix binned at 100 kb/500 kb resolution using R packages available at (https://bioconductor.org/packages/release/bioc/html/HiTC.html).^42^

*Identification of Chromatin Substructures—TADs*: The insulation score script described by (https://github.com/dekkerlab/cworld-dekker)^20^ was used to calculate insulation scores and insulation strength for each bin and identify TAD boundaries with insulation strength of pooled Hi‐C maps binned at 25 kb/40 kb resolution.

*Identification of Chromatin Substructures—Loops*: HiCCUPS script was applied for finding chromatin loops of pooled Hi‐C maps binned at 25 kb resolution (https://github.com/theaidenlab/juicer/wiki/HiCCUPS). To explore dynamics of chromatin loops across cell‐cycle progression, HiCCUPS Diff script (https://github.com/theaidenlab/juicer/wiki/HiCCUPSDiff) was used to detect differential loops between two successive subcycles, which appear in the first loop list and do not appear in the second one. The preprocessing scripts and the above two scripts were obtained from the Juicer package.^43^

*Aggregated Loop Analysis*: To calculate the average enrichment of a set of loops in a normalized Hi‐C contact map, the average of a series of submatrices derived from that contact map was calculated. Each of these submatrices was a 325 kb × 325 kb square centered at a single loop in the upper triangle of the contact map. The observed Hi‐C contact maps may be normalized by observed/expected (o/e) or Knight–Ruiz (KR).^44^ Each measurement shows a plot of an average matrix.

A set of loops corresponding to true peaks in a Hi‐C map should show prominent visual enrichment at the center of these plots. For the KR normalized contact map, the enrichment of a set of loops was evaluated by the ratio of the central pixel to the mean of the pixels in the lower left corner on the average matrix. For the o/e normalized contact map, the enrichment of a set of loops was evaluated by the ratio the central pixel to the mean of the pixels in the four corners on the average matrix.

*Comparison between the Enrichment of Two Set of Loops*: Having calculated two average matrices for two set of loops, the enrichment of two set of loops was compared. The ratio of the central pixel to the lower left corner of the two average matrices was computed into two submatrices, respectively. Furthermore, the Wilcoxon rank‐sum statistic was calculated for these two submatrices.

*Detecting Specific and Common TAD Boundaries with High Confidence*: To explore dynamics of TAD boundaries across cell‐cycle progression, TAD boundaries of high confidence were first kept with a uniform threshold across cell‐cycle progression, where insulation scores of boundaries was above the 90% quantile of those of all bins on Hi‐C contact maps across the whole substages. Then, adjacent TAD boundaries detected in different substages were merged, and the size of merged‐boundary was limited to less than the product of the number of substages and size of bins (i.e., 5 × 40 kb), whose insulation score was defined as the max of insulation scores of merged boundaries. Finally, the boundaries that appear in all substages were defined as common ones, and that appear in only one substage but not others as specific ones.

*Characterization of TAD Boundaries or Loops Relating to Genomic Features*: Genomic features, including 3′ UTR, 5′ UTR, exon, intron, and promoters (±2 kb around TSS), were first extracted from gene annotation file using R script (https://github.com/saketkc/gencode_regions/blob/master/create_regions_from_gencode.R). For a set of TAD boundaries, the number of genomic features overlapping with these boundaries was counted as observed scores. The expected score was counted by the number of genomic features overlapping with random boundaries, which were generated by selecting random regions of the corresponding chromosome for each observed boundary. Each random boundary should be the same size as its corresponding observed one. The fold change between observed and expected score was used to describe the enrichment of overlapping between TAD boundaries and genomic features. For a set of loops, each loop was transformed into two genomic regions. The calculation of enrichment for these genomic regions was the same to the one for TAD boundaries as above.

*Cell Cycle Annotated Genes*: Two lists of mouse cell‐cycle annotated genes were obtained from mouse genome informatics (MGI) (http://www.informatics.jax.org/). One list contains 1609 genes relating to cell‐cycle process and regulation, the other list contains 168 genes relating to cell‐cycle checkpoint (Tables S9 and S10, Supporting Information). The background gene set in differential loop analysis was downloaded from https://www.gencodegenes.org/mouse_releases/current.html.

*Distance Distribution from Gene Sets to Loop Sets*: For a given gene set and a loop set, the closest distance of each gene to the regions of loop sets was calculated, which characterized the distance distribution between these two sets.

*GO‐Term and PPI Network Enrichment Analysis*: Metascape, a gene annotation or analysis resource (http://metascape.org/gp/index.html#/main/step1), was applied for GO‐term enrichment analysis for a given mouse gene set. For a gene set overlapping with a differential loop set between two loop lists, all genes overlapping with the first loop list were used as background genes for GO‐term enrichment analysis. To conduct PPI network enrichment analysis for a given mouse gene set, and homologous ones in human of these genes were entered to Metascape to identify densely connected network components and enriched terms.

*Smoothing Contact Count Within Loops, Insulation Scores of Boundaries, and Gene Expression at Single‐Cell Resolution*: For a given loop, contact count within this loop was first normalized by the total contact count of corresponding chromosome for each single cell, and then the smoothed contact count was calculated by averaging contact count within the loop of 101 single cells centered at each single cell along inferred trajectory based on the single‐cell Hi‐C dataset.

For a given boundary, insulation score with window = 400 kb for each single cell (see “CIRCLET—Extracting Features” section) was first computed, and then the insulation score of each cell was smoothed as described above about loop contact count smoothing.

For a given gene, the smoothed gene expression was calculated by averaging values within the loop of 31 single cells centered at each single cell along the inferred trajectory based on the single cell RNA‐seq dataset.

*Identification of a Cell‐Cycle Trajectory Using a Single‐Cell Rna‐Seq Dataset*: A single‐cell RNA‐seq dataset consisting of 182 cells for G1, S, and G2/M phases was collected^41^ and the trajectory reconstruction step of CIRCLET was applied to obtain a reliable trajectory based on the cell‐cycle annotated genes (Figure S5, Supporting Information).

*Data Availability*: CIRCLET is a free, open source software under MIT License (OSI‐compliant), is implemented in python 3.6, and is freely available at https://github.com/zhanglabtools/CIRCLET or http://page.amss.ac.cn/shihua.zhang/software.html. All datasets in this manuscript are public datasets. Their available link addresses are placed.

## Conflict of Interest

The authors declare no conflict of interest.

## Supporting information

Supplementary
