# Supplementary material for: Circular Trajectory Reconstruction Uncovers Cell‐Cycle Progression and Regulatory Dynamics from Single‐Cell Hi‐C Maps
Source: Adv Sci (Weinh). 2019 Sep 30;6(23):1900986. doi: 10.1002/advs.201900986 (PMC6891923; doi:10.1002/advs.201900986)
Supplement: Supplementary file 1 — Supplementary [file ADVS-6-1900986-s001.pdf]

Copyright WILEY-VCH Verlag GmbH & Co. KGaA, 69469 Weinheim, Germany, 2019.

ADVANCED SCIENCE Open Access

Supporting Information

for *Adv. Sci.,* DOI: 10.1002/advs.201900986

Circular Trajectory Reconstruction Uncovers Cell-Cycle Progression and Regulatory Dynamics from Single-Cell Hi-C Maps

Yusen Ye, Lin Gao, and Shihua Zhang**

Supplementary Information for

Circular trajectory reconstruction uncovers cell-cycle progression and regulatory dynamics from single-cell Hi-C maps

Yusen Ye[1], Lin Gao[1,*], and Shihua Zhang[2,3,4*]

[1] School of Computer Science and Technology, Xidian University, Xi’an 710071, Shaanxi, China;

[2] NCMIS, CEMS, RCSDS, Academy of Mathematics and Systems Science, Chinese Academy of Sciences, Beijing 100190, China;

[3] School of Mathematical Sciences, University of Chinese Academy of Sciences, Beijing 100049, China;

[4] Center for Excellence in Animal Evolution and Genetics, Chinese Academy of Sciences, Kunming 650223, China.

*Correspondence should be addressed to S.Z. (zsh@amss.ac.cn) or L.G. (lgao@mail.xidian.edu.cn).

Supplementary Table of Contents

I. Supplementary Methods

II. Supplementary Figures

III. Supplementary Tables

IV. Supplementary References

Supplementary Methods

Selection of features

We first normalized all features by min-max normalization into [0.01, 1]. We further filtered out some features by the variance (variance>0.04 for PCC, variance>0.015 for MCM). Other feature sets were selected in an inverse way.

Generating non-branch and cyclic trajectory

Based on the pseudo trajectory reconstructed by CIRCLET and tSNE map from four sets of cells (G1, ES, MS, LS/G2) labelled by FACS, noted as “Raw group”, we removed a union of cells in the head and tail of the pseudo trajectory and cells in tSNE map surrounded by red dotted boxes in Supplementary Figure S2A. The number of removed cells in the head and tail of the pseudo trajectory accounts for 5% of the whole cells, respectively. Thus, the above cell set, noted as “Removed group” or reduced group, can be used as input for reconstructing a non-branch trajectory ordering. Furthermore, we extended the reduced group by adding synthetic nodes to the location between the head and tail of non-branch trajectory to obtain a new set, noted as “Extended group”. Specifically, we first applied Principal Component Analysis (PCA) to map original feature space onto the top K principle components, assuming the smaller components representing noise, expressed as:

$$Z = U_K^T \times X ,$$

where $X \in \mathbb{R}^{N \times M}$ represents M samples with N features in original space and $U_K^T \in \mathbb{R}^{K \times N}$ is loadings and $Z \in \mathbb{R}^{K \times M}$ is the top K eigenvectors after dimension reduction (default $K = 5$). We next selected two sets as seeds at both ends of the non-branch trajectory surrounded by red dotted boxes in tSNE map (Supplementary Figure S2B, left panel) and iteratively extended this cell set in a low-dimensional space. We obtained a synthetic node z_{syn}^l by computing a weighted average of randomly 20 cells from two seed sets (10 cells per set), expressed as $Z_{RS} = [z_{RS}^1, z_{RS}^2, \cdots, z_{RS}^{20}]$. The weighted average is calculated by

$$z_{syn}^l = Z_{RS} * w^T, \quad |w| = 1,$$

where w^T is random weight vector for Z_{RS}. Repeating the above process to obtain 20 synthetic nodes and adding these synthetic nodes to both two seed sets, which are seed cells of next iteration. Iteratively running the above processes 10 times, we added 200 synthetic nodes labelled by gray between the head and tail of a non-branch trajectory (Figure 3A, bottom panel). Finally, we mapped the low-dimensional synthetic nodes Z_{syn} back to the original space by loadings U_K^T, expressed as

$$X_{syn} = U_K \times Z_{syn}.$$

“Extended group” is used as input for reconstructing a cyclic trajectory.

Computational resource and complexity

CIRCLET is implemented under computer environment - inter core 3.4GHz and 128G RAM). It consists of four different feature sets from 1173 single cell Hi-C maps: MCM, CDD, Ins and PCC. The size of input matrix is approximately 8561×1173 by choosing significant features (Materials and Methods), where 8561 is the number of features, and 1173 is the number of single cells. CIRCLET applies the diffusion map method to reduce high-dimension space to low-dimension space. The time complexity of it is $O\left(N^3\right)$, where N is the number of cells in the trajectory. Next, CIRCLET constructs KNN graph and computes initial ordering from a starting cell, whose complexity are $O\left(N^2\right)$ and $O\left(N^2 * \log(N)\right)$ respectively. CIRCLET chooses a series of cells as "waypoints", whose number is a constant $W\left(50 \le W \le 250\right)$. Note that the results are not sensitive to W. Then, CIRCLET detects the orientation of cells and refines the ordering of cells, whose complexity are $O\left(N^2 * \log(N)\right)$ and $O(N)$ respectively.

Waypoints

CIRCLET selects a series of cells called waypoints along the whole trajectory to provide sparse approximation for the entire dataset. Since random sampling of cells can result in outliers as waypoints, waypoints are selected by a median filter strategy to preventing the outlier cells. For each randomly selected cell, we identify its k nearest neighbors and define one cell closest to the median of all neighbors as a waypoint.

Note the initial ordering of cells is relatively susceptible to noise as the distance increases. CIRCLET addresses this issue by sampling a series of waypoints to guide the cell ordering. Sampling waypoints helps to order cells by averaging across the waypoints. Meanwhile, the closer waypoints from the source cell will give a relatively higher weight. Furthermore, disagreements between the perspectives of waypoints are used to split the cyclic trajectory into two semicircles of opposite directions.

Supplementary Figures

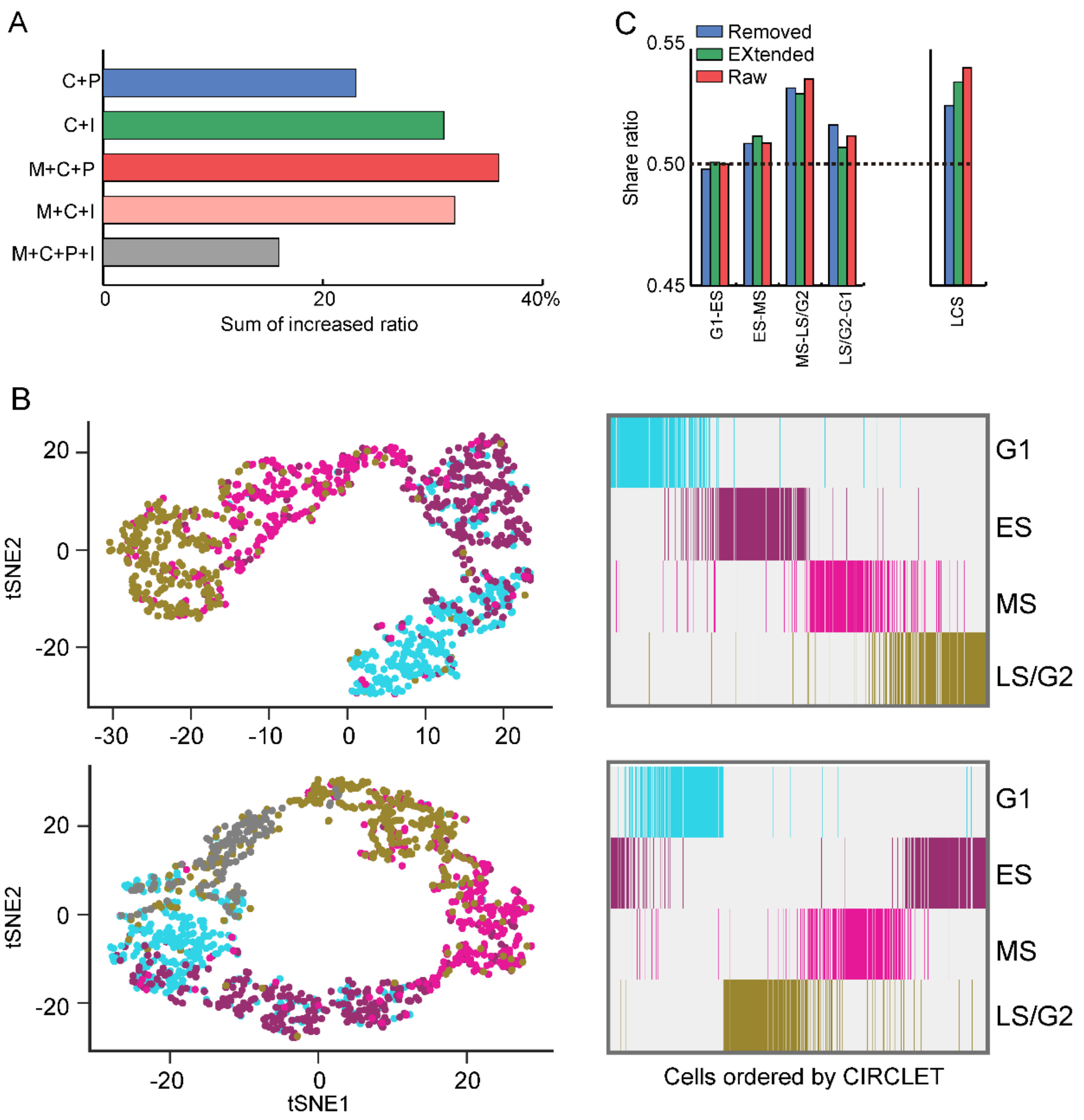

Figure S1. Exploring the preference of multiple feature sets and their combinations. (A) Sum of increased ratio of CIRCLET compared to the competing study (Nagano *et al.*) using the above five combinations of feature sets under five evaluation indexes. (B) tSNE maps and corresponding reconstructed cell-cycle trajectories by CIRCLET from four FACS-sorted cells (G1, ES, MS, LS/G2) based on the best combination (MCM+CDD+PCC). Two groups of single cells are analyzed. One is the cell set removing the part of cell-cycle beginning and end phasing cells from the inferred sequence of raw sets; and the other is the cell set extending simulated cells between cell-cycle beginning and end phasing from removed ones. (C) The share ratio of five scores between the reconstructed trajectory by Nagano *et al.* and CIRCLET on the best combination of feature sets for raw sets, reduced sets and extended sets respectively. These evaluation indexes include AUC scores between two consecutive cell-cycle phases (denoted as G1-ES, ES-MS, MS-LS/G2 and LS/G2-G1) and LCS for measuring labels change of consecutive cells on the entire reconstructed trajectory.

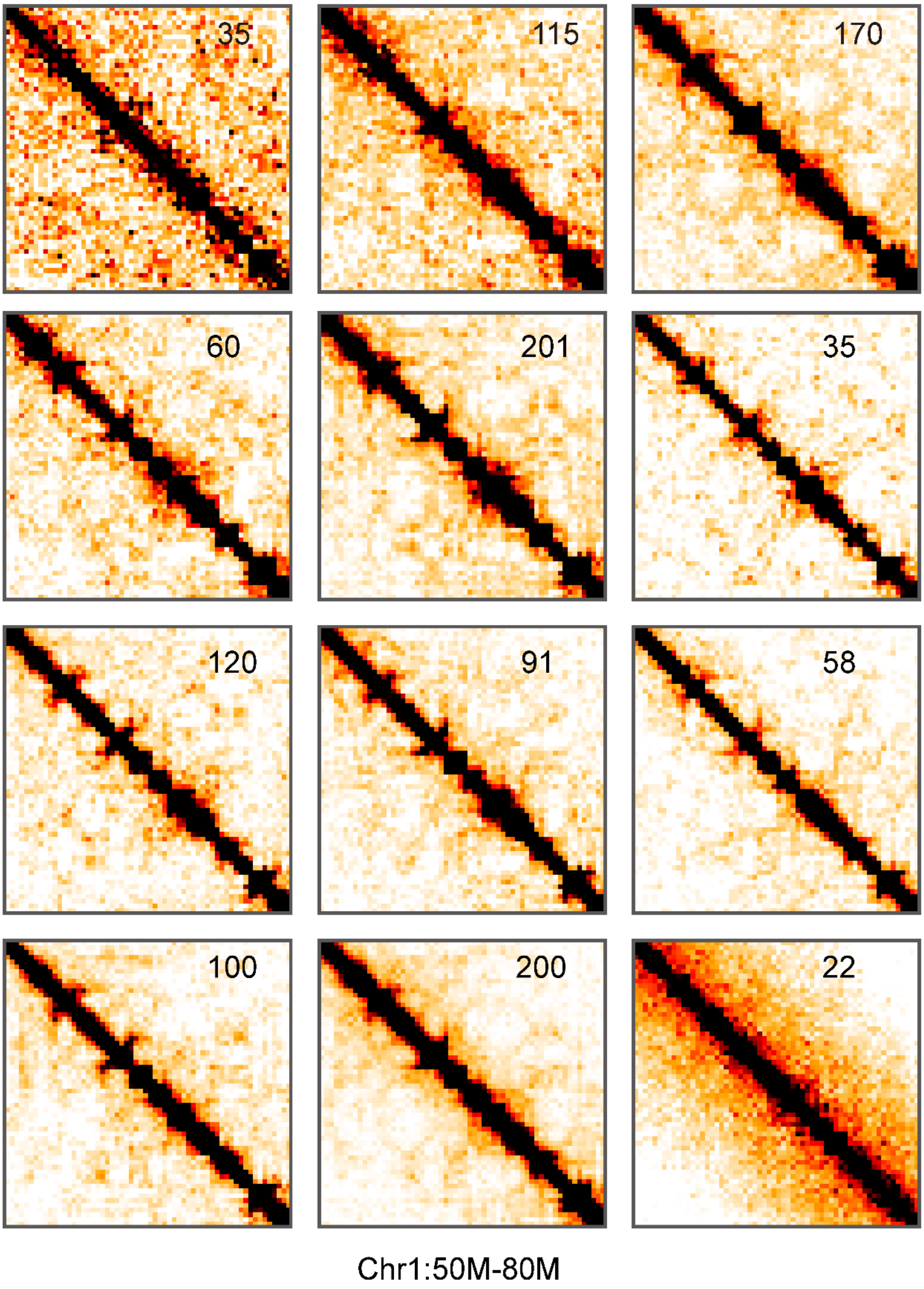

Figure S2. Chromatin interaction maps binned at 500kb for Chr1:50-80M region across 12 different sub-cycles. The number on each map indicates the number of single cells in this sub-cycle.

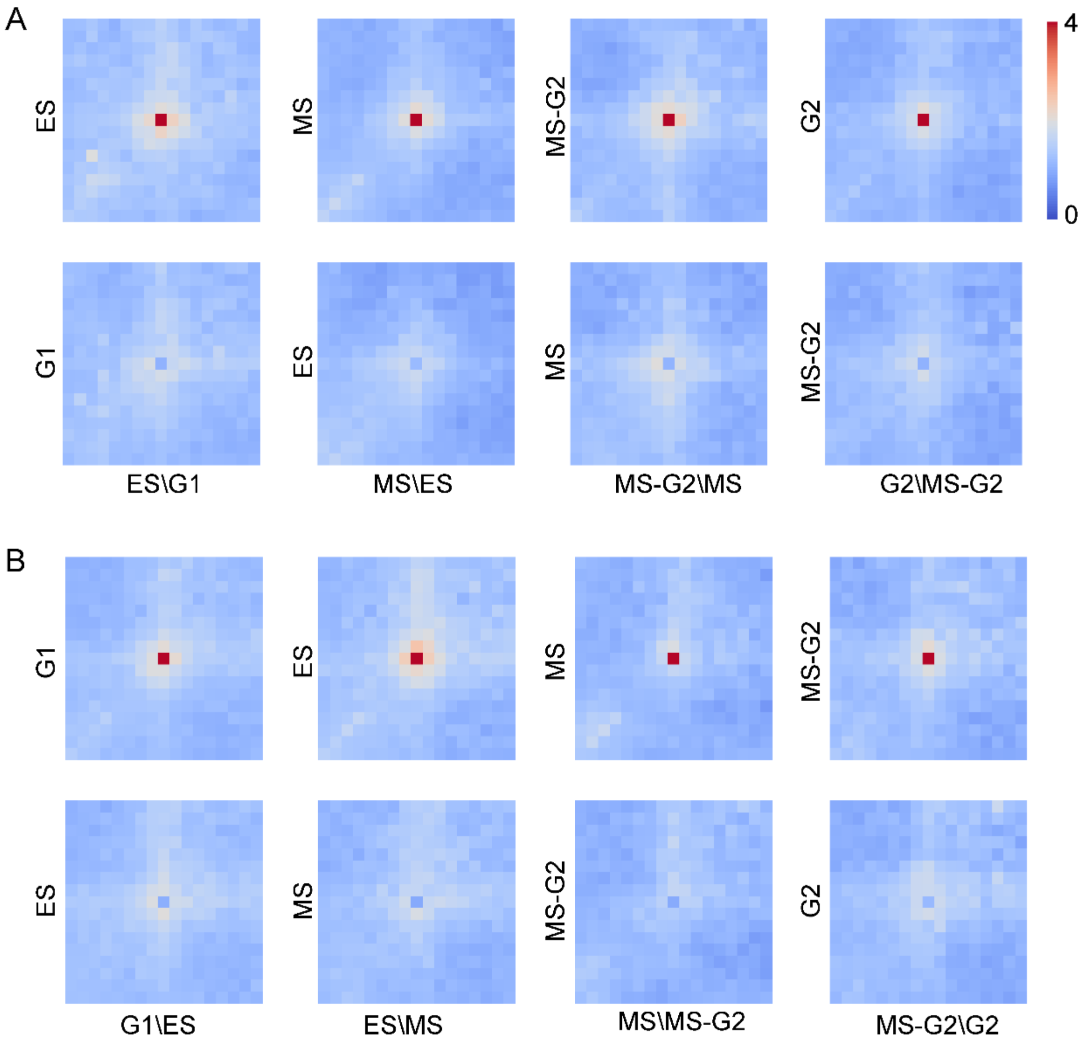

Figure S3. **The plot of differential loops across cell cycle phases.** (A) Average Hi-C maps around differential loops between two consecutive phases across cell cycle. These loops appear in the first loop list, but not in the second loop list (e.g. appearing in ES, but not in G1 for ES\G1, The corresponding two maps are derived from contact maps of ES and G1 sub-cycles, respectively). (B) Average Hi-C maps around differential loops between consecutive phases against cell cycle.

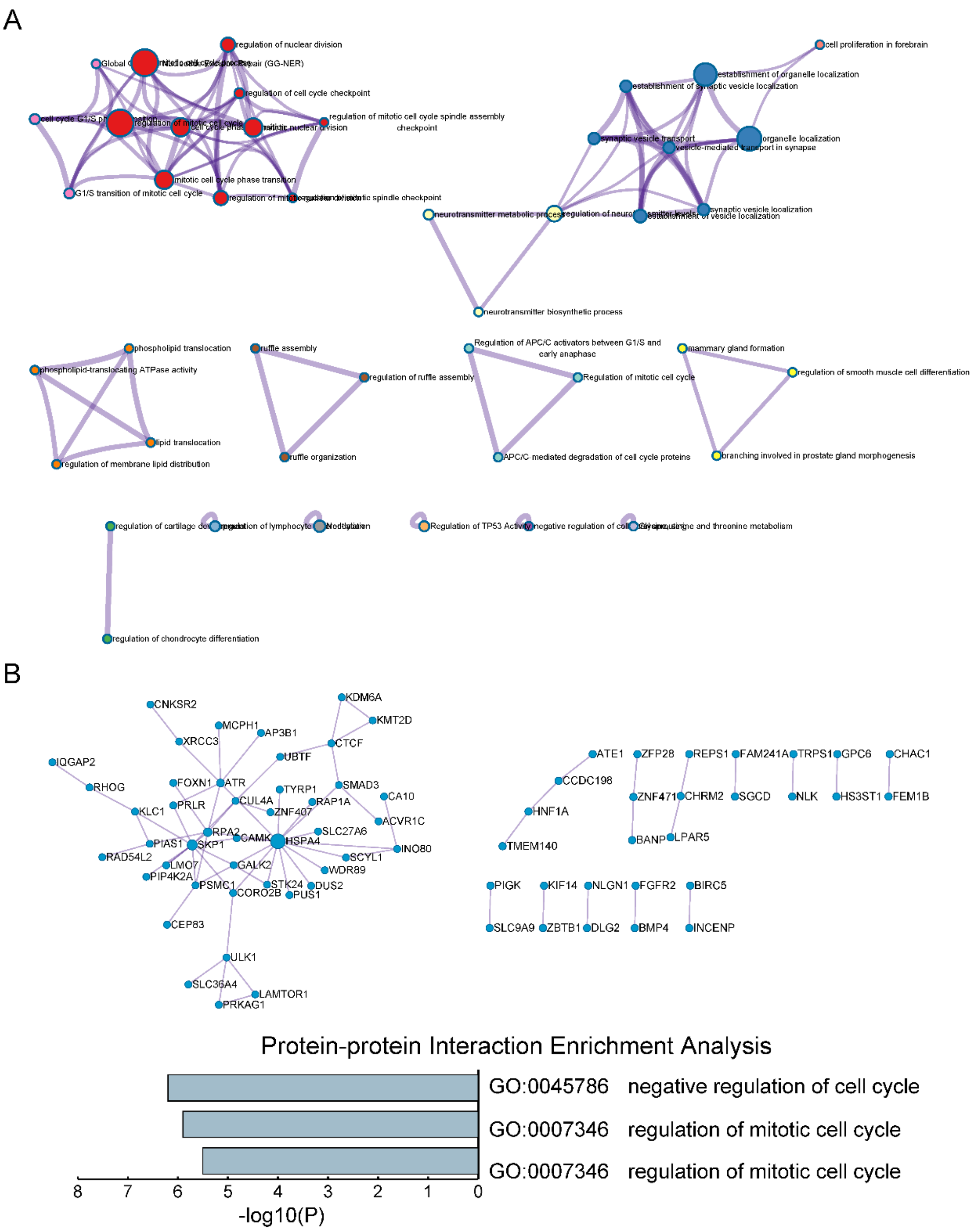

Figure S4. (A) Functional enrichment analysis of genes relating to differential loops between ES and G1. These loops are observed in ES phase, but not in G1 phase. (B) PPI network enrichment analysis of human homologous gene set mapped from mouse genes relating to differential loops between ES and G1. These two networks in (A) and (B) relate to Figure 4E.

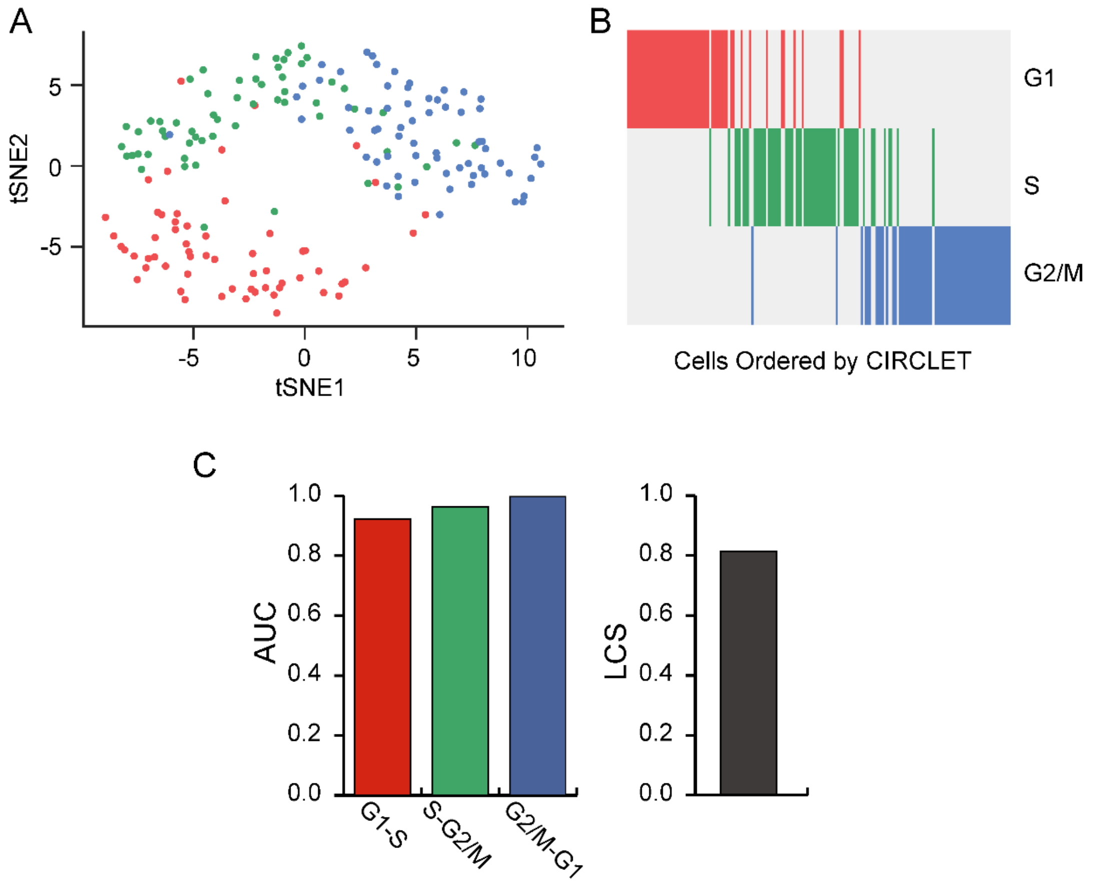

Figure S5. (A-B) tSNE map and reconstructed cell-cycle trajectory by CIRCLET from four FACS-sorted cell phases (G1, S, G2/M) based on 959 cell-cycle annotated genes from a single-cell RNA-seq dataset of 182 cells. (C) Comparison of four evaluation indexes for the reconstructed cell-cycle trajectory by CIRCLET based on this single-cell RNA-seq dataset. These scores consist of three AUC scores, LCS.

Supplementary Tables

Table S1. The number (#) of loops and their overlapping genes in different sub-stages.

	**G1**	**ES**	**MS**	**MS-G2**	**G2**
#loops identified by HiCCUPS [1]	3694	2361	1759	2007	2811
#genes overlapping with loops	4318	3292	2766	2918	3697

Table S2. The number (#) of TAD boundaries, TAD boundaries of high confidence, specific TAD boundaries, specific TAD boundaries overlapping with background genes and overlapping genes of specific TAD boundaries.

	**G1**	**ES**	**MS**	**MS-G2**	**G2**
#TAD boundaries identified by insulation score script [2]	2923	2828	2892	2933	3008
#TAD boundaries with the top 10% insulation scores for all cell-cycle phases	842	1074	836	777	741
#specific TAD boundaries across all cell-cycle phases	98	214	115	60	49
#specific TAD boundaries overlapping with gene set from gencode.vM9	86	184	99	46	43
#genes overlapping with specific TAD boundaries	215	511	236	119	116

Table S3. The number (#) of differential loops and their overlapping genes between continuous phases across cell cycle. These loops appear in the first loop list, but not in the second loop list (e.g. appear in ES, but not in G1 for ES\G1).

	**ES\G1**	**MS\ES**	**MS-G2\MS**	**G2\MS-G2**
#differential loops between different phases	172	129	99	118
#gene associated with differential loops	349	301	218	231

Table S4. The number (#) of differential loops and their overlapping genes between adjacent phases against cell cycle.

	**MS-G2\G2**	**MS\MS-G2**	**ES\MS**	**G1\ES**
#differential loops between different phases	67	79	233	363
#gene associated with differential loops	153	179	424	698

Table S5-S8. Enriched GO-terms of genes overlapping with differential loops between ES and G1, MS and ES, MS-G2 and MS, G2 and MS-G2 respectively.

Table S9. Genes related to cell cycle progress and regulation from MGI database.

Table S10. Genes related to cell cycle checkpoint from MGI database.

Table S11. Multiple composite metrics (MCM) defined in [3].

Metrics	Explanation
**% near**	percentage of contacts in bins 38-89 out of all valid bins (38-89: 26.9kb-2233kb)
**% mitotic**	percentage of contacts in bins 90-109 out of all valid bins (2435kb-12633kb)
**farAvgDist**	mean contact distance considering bins >= 98 (4870kb)
**rawRepliScore**	fraction of early-replicating fends out of all fends in the contact map
**Trans contacts**	percentage of contacts between different chromosomes
**Trans_align**	The authors extracted trans-chromosomal contacts and scaled the coordinates of the contacting fends by the length of their respective chromosomes. Alignment was then approximated using the Pearson correlation between the two scaled fend coordinate vectors
**cis_ab_comp/trans_ab_comp**	The compartment score of a cell is the depletion of A-B contacts over the count expected by the marginal distribution of A or B contacts
**mean_insu**	Domain borders were called from the insulation profile of the pooled map by identifying highly insulating regions between domains (insulation above the 90% quintile) and selecting in each element the 1KB with highest insulation score. To calculate the cell mean insulation over a set of borders (either all borders), the total number of border violating contacts is compared to the total number of contacts around the border
**loop_enr_EE/ loop_enr_LL**	Loop foci enrichment quantifies how concentrated contacts are around a loop. We calculate the ratio between contacts in a small window (20x20kb) centered on the loop and contacts in a larger window (60x60kb), normalizing by the expected ratio if contacts were uniformly distributed (1/9). To get a mean loop foci enrichment over a group of loops, the sum of contacts in all small windows is compared with the sum of contacts in the larger ones

Table S12. Important regulatory genes overlapping with differential loops across cell cycle progression.

Table S13. Important regulatory genes overlapping with common and specific TAD boundaries across cell cycle progression.

Supplementary References

1. Rao SS, Huntley MH, Durand NC, Stamenova EK, Bochkov ID, Robinson JT, Sanborn AL, Machol I, Omer AD, Lander ES: **A 3D map of the human genome at kilobase resolution reveals principles of chromatin looping.** *Cell* 2014, **159:**1665-1680.
2. Crane E, Bian Q, McCord RP, Lajoie BR, Wheeler BS, Ralston EJ, Uzawa S, Dekker J, Meyer BJ: **Condensin-driven remodelling of X chromosome topology during dosage compensation.** *Nature* 2015, **523:**240.
3. Nagano T, Lubling Y, Várnai C, Dudley C, Leung W, Baran Y, Mendelson Cohen N, Wingett S, Fraser P, Tanay A: **Cell-cycle dynamics of chromosomal organization at single-cell resolution.** *Nature* 2017, **547:**61-67.
